# Treatment Site Does Not Affect Changes in Pulse Wave Velocity but Treatment Length and Device Selection Are Associated With Increased Pulse Wave Velocity After Thoracic Endovascular Aortic Repair

**DOI:** 10.3389/fphys.2021.739185

**Published:** 2021-10-22

**Authors:** Daijiro Hori, Tomonari Fujimori, Sho Kusadokoro, Takahiro Yamamoto, Naoyuki Kimura, Atsushi Yamaguchi

**Affiliations:** Department of Cardiovascular Surgery, Saitama Medical Center, Jichi Medical University, Saitama, Japan

**Keywords:** pulse wave velocity, endovascular treatment, aortic arch aneurysm, descending thoracic aortic aneurysm, treatment length, Najuta, arterial compliance, TEVAR

## Abstract

**Background:** Endovascular treatment of aortic aneurysm is associated with an increase in pulse wave velocity (PWV) after surgery. However, the effect of different types of endovascular devices on PWV at different sites of the thoracic aorta remains unclear.

**Objectives:** The purposes of this study were (1) to investigate the changes in PWV after endovascular treatment of thoracic aortic aneurysm; (2) to evaluate whether there is a difference in the changes in PWV at different treatment sites; and (3) to evaluate the effect of treatment length on changes in PWV.

**Methods:** From July 2008 to July 2021, 276 patients underwent endovascular treatment of the true thoracic aortic aneurysm. Of these patients, 183 patients who underwent preoperative and postoperative PWV measurement within 1 year of surgery were included in the study. The treatment length index was calculated by treatment length divided by the height of the patients.

**Results:** Five different types of endovascular devices were used (Najuta, Kawasumi Laboratories, Inc., Tokyo, Japan; TAG, W.L. Gore & Associates, Inc., AZ, USA; Relay, Bolton Medical, Inc., FL, USA; Talent/Valiant, Medtronic, MN, USA; and Zenith, Cook Medical, IN, USA). There was no significant change in PWV in patients receiving Najuta (Before: 2,040 ± 346.8 cm/s vs. After: 2,084 ± 390.5 cm/s, *p* = 0.14). However, a significant increase was observed in other devices: TAG (Before: 2,090 ± 485.9 cm/s vs. After: 2,300 ± 512.1 cm/s, *p* = 0.025), Relay (Before: 2,102 ± 465.3 cm/s vs. After: 2,206 ± 444.4 cm/s, *p* = 0.004), Valiant (Before: 1,696 ± 330.2 cm/s vs. After: 2,186 ± 378.7 cm/s, *p* < 0.001), and Zenith (Before: 2,084 ± 431.7 cm/s vs. After: 2,321 ± 500.6 cm/s, *p* < 0.001). There was a significant increase in PWV in patients treated from aortic arch (Before: 2,006 ± 333.7 cm/s vs. After: 2,132 ± 423.7 cm/s, *p* < 0.001) and patients treated from descending thoracic aorta (Before: 2,116 ± 460.9 cm/s vs. After: 2,292 ± 460.9 cm/s, *p* < 0.001). Multivariate analysis showed that treatment site was not an independent factor associated with changes in PWV. However, Najuta (Coef −219.43, 95% CI −322.684 to −116.176, *p* < 0.001) and treatment index (Coef 147.57, 95% CI 24.826 to 270.312, *p* = 0.019) were independent factors associated with changes in PWV.

**Conclusion:** Najuta did not show a significant increase in PWV, while other commercially available devices showed a significant increase. The treatment site did not have a different effect on PWV. However, the treatment length was an independent factor associated with an increase in PWV.

## Introduction

Pulse wave velocity (PWV), which is a measure for aortic stiffness, is a well-known factor associated with cardiovascular events (Sutton-Tyrrell et al., 2005; Mattace-Raso et al., 2006; Munakata, 2016; Tomiyama et al., 2016; Ohkuma et al., 2017; Takae et al., 2019). With atherosclerotic changes of the aorta, PWV increases, resulting in reduced diastolic pressure and reduced coronary perfusion. Furthermore, excess volume overload to the peripheral causes end-organ damages (Spadaccio et al., 2016; Tomiyama et al., 2016). Increase in PWV is also observed in patients undergoing graft replacement (Spadaccio et al., 2016). A similar phenomenon was also observed after endovascular treatment of abdominal aortic aneurysms which resulted in cardiac hypertrophy (Takeda et al., 2014). Changes in cardiac dimension have also been reported in patients who underwent endovascular treatment for the thoracic aortic arch aneurysm (Hori et al., 2020).

Meanwhile, *in vitro* study of abdominal aortic endoprostheses on PWV has shown that endoskeleton type stent graft had less effect on PWV compared with other devices (van Noort et al., 2018). Furthermore, medications such as vasodilators and anti-inflammatory drugs have been suggested to reduce PWV (Janic et al., 2014).

Pulse wave velocity is regulated by aortic compliance, and the aortic arch is known to contribute to ~50% of the total arterial compliance (Kanzaki, 2014). The purposes of this study were (1) to investigate the changes in PWV after endovascular treatment of thoracic aortic aneurysm using commercially available endoprosthesis; (2) to evaluate whether there is a difference in the changes in PWV at different treatment sites of the thoracic aorta; and (3) to evaluate the effect of treatment length on changes in PWV. Our primary hypothesis is that Najuta, which is an endoskeleton type endoprosthesis, has less effect on PWV compared with other commercially available exoskeleton type endoprostheses. The secondary hypothesis is that endoprosthesis placement in the descending thoracic aorta will lead to less increases in postoperative PWV compared with endoprosthesis placement in the aortic arch. Another secondary hypothesis is that longer treatment length would lead to higher increases in postoperative PWV.

Although the main goal of aortic aneurysm repair is to prevent aneurysmal rupture, physiological changes after endovascular treatment should also be considered. Modifying treatment strategy to minimize the changes in PWV may provide better long-term outcomes in patients undergoing endovascular treatment for thoracic aortic aneurysms.

## Materials and Methods

From July 2008 to July 2021, 276 patients underwent endovascular treatment of the true thoracic aortic aneurysm. Patients with ABI < 0.9 were excluded from the study. Of the remaining patients, 183 patients who underwent PWV measurement within 1 year of surgery were included. Changes in PWV before and after surgery were evaluated for different types of endoprostheses. Furthermore, treatment site and treatment length were evaluated for their effects on the degree of changes in PWV. The study was approved by the institutional review board of Saitama Medical Center, Jichi Medical University (S20-205).

### Surgical Strategy

Choice of endoprosthesis was selected by the preference of the surgeon. All endovascular treatments were performed under general anesthesia. For patients receiving Najuta (Kawasumi, Japan), tug of wire technique was prepared from the right brachial artery to the femoral artery (Yuri et al., 2013). Najuta device was introduced from the femoral artery and was guided with a wire to the ascending aorta. The device was deployed without the use of control pacing or mechanical cardiac support. For patients requiring debranching, bypass surgery was performed prior to stent graft deployment. The orifice of each debranched artery was occluded with a coil or a stitch.

For patients receiving other commercially available endoprostheses, the device was guided into the aorta using a stiff wire. Similar to Najuta, the device was deployed without the use of control pacing and mechanical cardiac support.

### Pulse Wave Velocity

Brachial-ankle PWV was measured in awake patients, before and after surgery, using a non-invasive device (Omron Colin, Tokyo, Japan) in the physiology laboratory by a trained technician. Two pressure-sensitive transducers were placed on the arm and the ankle. Simultaneous recording of the pulse waves at two different points was performed. The pulse transit time and the distance between the transducers over the body surface were assessed to calculate the PWV [PWV (cm/s) ¼ travel distance (cm) /transit time (s)] (Munakata, 2016). PWV measurements within 1 year of surgery were collected, and measurements with closest blood pressure before and after surgery were used for the analysis.

### Data Analysis

The normal distribution of the data was evaluated using the Kolmogorov–Smirnov test. For continuous variables that were normally distributed, Student's *t*-test (mean ± standard deviation) was used for comparing two groups, paired *t*-test (mean ± standard deviation) was used for comparing paired data, and one-way ANOVA (mean ± standard deviation) was used for comparing more than three groups. For continuous variables that were not normally distributed, Mann–Whitney U test (median, Q1–3) was used for comparing two groups, and Kruskal–Wallis test (median, Q1–3) was used for comparing more than three groups. For categorical variables, Fisher's exact test (*n*, %) was used. Evaluation of correlation between two continuous variables was performed by Pearson's correlation test. Changes in PWV before and after surgery were evaluated for each endoprosthesis using paired *t*-test. Patients were divided into two groups, i.e., those who were treated from the aortic arch and those who were treated from the descending thoracic aorta. Demographics and changes in PWV were evaluated for each group. Factors associated with the degree of changes in PWV were evaluated by multivariate analysis for observed differences in PWV after surgery. Possible confounding factors, such as age, gender, medication for hypertension, diabetes, and dyslipidemia, were included in the linear regression model for the evaluation of the type of endoprosthesis, treatment site, and treatment length in association with an increase in PWV. The treatment length index was calculated by treatment length divided by the height of the patients. The *p* < 0.05 were considered statistically significant. The analysis was performed using Stata (version 13.1, Stata Corp, College Station, TX, USA) and Prism (version 8.0.2, GraphPad Software Inc., La Jolla, CA, USA).

### Sample Size

Sample size calculation was performed based on the data from our previous study (Hori et al., 2020). The difference in the mean PWV before and after surgery for patients undergoing endovascular treatment for aortic arch aneurysm was 125 cm/s, while the standard deviation of PWV for both measurements was 421 cm/s. From these data, a sample size of 72 patients would provide a statistical evaluation with α = 0.05 and a power of 0.80. A sample size of 66 patients would provide a statistical power of 0.77; and a sample size of 76, 107, and 117 patients would provide a statistical power of 0.82, 0.92, and 0.94, respectively.

## Results

Five different types of endovascular devices were used (Najuta, Kawasumi Laboratories, Inc., Tokyo, Japan: *n* = 66; TAG, W.L. Gore & Associates, Inc., AZ, USA: *n* = 20; Relay, Bolton Medical, Inc., FL, USA: *n* = 20; Talent/Valiant, Medtronic, MN, USA: *n* = 30; Zenith, Cook Medical, IN, USA: *n* = 47). Angiotensin receptor blocker was more often used in patient receiving non-Najuta devices (Najuta: 27.3% vs. non-Najuta: 43.6%, *p* = 0.039). Patients treated from aortic arch were more often observed in patients receiving Najuta (Najuta: 89.4% vs. non-Najuta: 41.0%, *p* < 0.001), and the treatment index was longer in patients receiving Najuta (Najuta: 1.38 ± 0.340 vs. non-Najuta: 1.20 ± 0.350, *p* = 0.001) (Table 1).

**Table 1:** Demographics of the patients included in the study.

		**Najuta vs. non-Najuta**			**Non-Najuta device**				
	**Total**	**Najuta**	**Non-Najuta**	***p***	**TAG**	**Relay**	**Valiant**	**Zenith**	***p***
	***n* = 183**	***n* = 66**	***n* = 117**	**Najuta vs. non-Najuta**	***n* = 20**	***n* = 20**	***n* = 30**	***n* = 47**	**All device**
Age (years)	74 ± 7.8	75 ± 7.8	74 ± 7.8	0.71	73 ± 7.4	76 ± 10.2	75 ± 7.3	74 ± 7.3	0.69
Male (%)	139 (76.0%)	53 (80.3%)	86 (73.5%)	0.37	14 (70.0%)	16 (80.0%)	20 (66.7%)	36 (76.6%)	0.61
Creatinine (mg/dl)	0.97 (0.77 to 1.29)	0.87 (0.76–1.16)	1.0 (0.79–1.33)	0.15	0.93 (0.79–1.12)	0.96 (0.74–1.34)	0.89 (0.75–1.30)	1.01 (0.87–1.40)	0.46
Hemoglobin (g/dl)	12.4 ± 1.77	12.4 ± 1.65	12.5 ± 1.84	0.75	12.8 ± 1.68	12.2 ± 1.74	12.4 ± 1.75	12.5 ± 2.04	0.87
Hypertension (%)	173 (94.5%)	63 (95.5%)	110 (94.0%)	1	17 (85.0%)	18 (90.0%)	29 (96.7%)	46 (97.9%)	0.19
Dyslipidemia (%)	85 (46.4%)	32 (48.5%)	53 (45.3%)	0.76	8 (40.0%)	7 (35.0%)	15 (50.0%)	23 (48.9%)	0.79
Diabetes (%)	41 (22.4%)	16 (24.2%)	25 (21.4%)	0.71	5 (25.0%)	1 (5.0%)	8 (26.7%)	11 (23.4%)	0.35
Chronic obstructive pulmonary disease (%)	23 (12.6%)	7 (10.6%)	16 (13.7%)	0.65	3 (15.0%)	2 (10.0%)	6 (20.0%)	5 (10.6%)	0.71
Cerebral vascular disease (%)	28 (15.3%)	11 (16.7%)	17 (14.5%)	0.83	3 (15.0%)	2 (10.0%)	2 (6.7%)	10 (21.3%)	0.5
Ischemic heart disease (%)	44 (24.0%)	18 (27.3%)	26 (22.2%)	0.47	5 (25.0%)	5 (25.0%)	6 (20.0%)	10 (21.3%)	0.94
Tobacco user (%)	137 (74.9%)	51 (77.3%)	86 (73.5%)	0.6	14 (70.0%)	15 (75.0%)	23 (76.7%)	34 (72.3%)	0.95
Angiotensin receptor blocker (%)	69 (37.7%)	18 (27.3%)	51 (43.6%)	0.039	10 (50.0%)	8 (40.0%)	14 (46.7%)	19 (40.4%)	0.22
Calcium channel blocker (%)	128 (69.9%)	45 (68.2%)	83 (70.9%)	0.74	12 (60.0%)	17 (85.0%)	21 (70.0%)	33 (70.2%)	0.52
Beta blocker (%)	78 (42.6%)	25 (37.9%)	53 (45.3%)	0.35	11 (55.0%)	9 (45.0%)	13 (43.3%)	20 (42.6%)	0.75
HMG-CoA inhibitor (%)	92 (50.3%)	31 (47.0%)	61 (52.1%)	0.54	10 (50.0%)	8 (40.0%)	17 (56.7%)	26 (55.3%)	0.73
Arch treatment (%)	107 (58.5%)	59 (89.4%)	48 (41.0%)	<0.001	7 (35.0%)	17 (85.0%)	8 (26.7%)	16 (34.0%)	<0.001
Treatment length (mm)	202.9 ± 55.81	223.1 ± 49.29	192.0 ± 56.28	<0.001	195.5 ± 63.18	198.4 ± 68.86	196.5 ± 60.89	184.5 ± 43.39	0.008
Treatment index (mm/cm)	1.26 ± 0.357	1.38 ± 0.340	1.20 ± 0.350	0.001	1.24 ± 0.434	1.22 ± 0.406	1.22 ± 0.376	1.15 ± 0.259	0.016
Difference in PWV (cm/s)	147 ± 326.0	44 ± 322.0	204 ± 315.1	0.001	210 ± 447.6	104 ± 157.0	217 ± 345.3	237 ± 276.9	0.012
Difference in sBP (mmHg)	−1 ± 14.8	−2 ± 12.2	−1 ± 16.2	0.67	−5 ± 21.0	−9 ± 11.4	1 ± 15.5	4 ± 14.6	0.012

*sBP, systolic blood pressure*.

*p-value for all devices, including Najuta device*.

**Figure 1:**
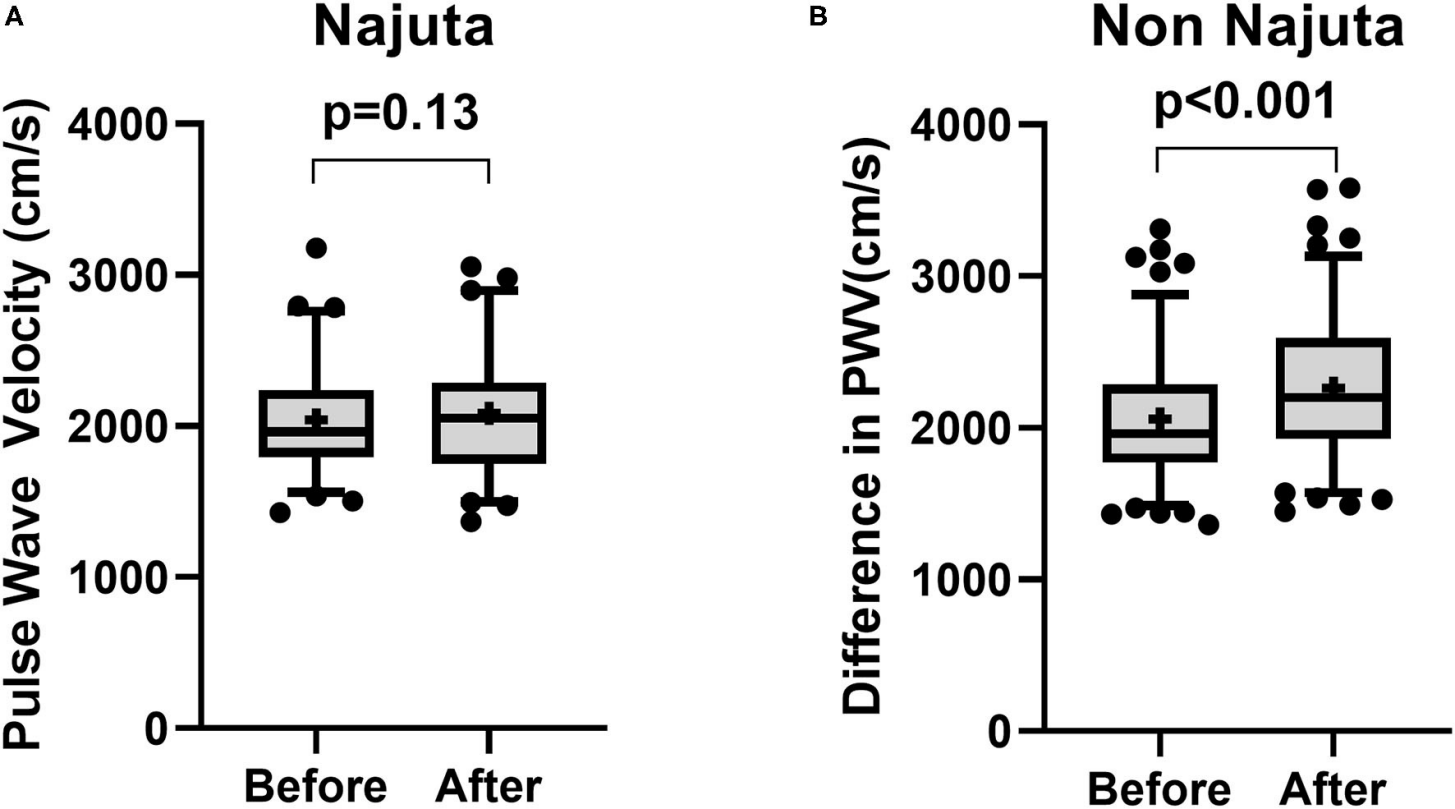
Box and Whisker plot for pulse wave velocity before and after surgery for patients receiving Najuta device **(A)** and non-Najuta device **(B)**. There was no significant difference in pulse wave velocity in patients receiving Najuta device **(A)**. There was a significant increase in pulse wave velocity in patients receiving non-Najuta devices **(B)**. Whisker shows 5–95 percentile range and + shows the mean value.

**Figure 2:**
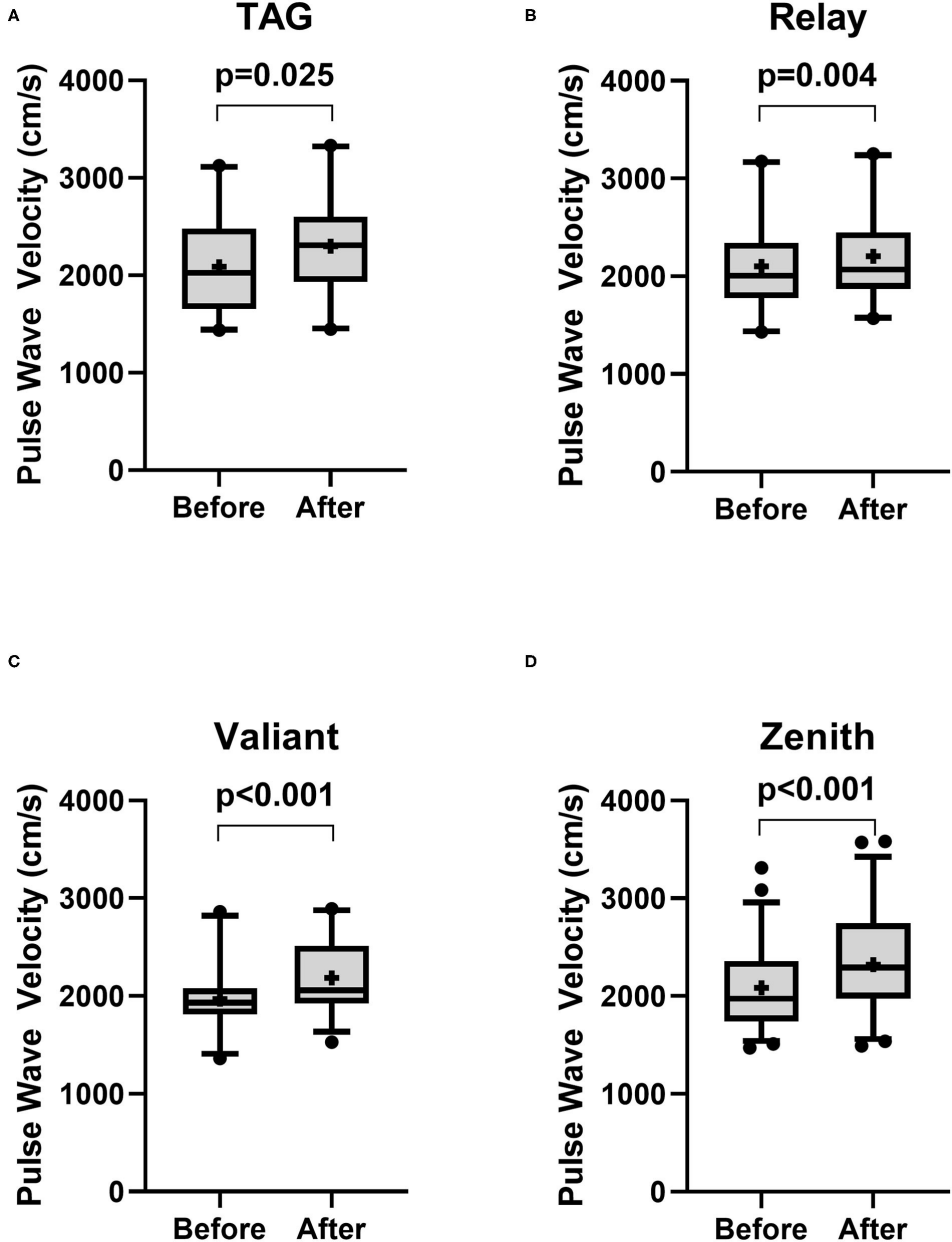
Box and Whisker plot for pulse wave velocity before and after surgery for patients receiving non-Najuta devices. There was a significant difference in pulse wave velocity in patients receiving TAG **(A)**, Relay **(B)**, Valiant **(C)**, and Zenith **(D)**. Whisker shows 5–95 percentile range and + shows the mean value.

There was no significant change in PWV in patients receiving Najuta (Before: 2,040 ± 346.8 cm/s vs. After: 2,084 ± 390.5 cm/s, *p* = 0.14, *n* = 66) (Figure 1A). However, a significant increase was observed in patients receiving non-Najuta devices (Before: 2,058 ± 422.1 cm/s vs. After: 2,263 ± 462.8 cm/s, *p* < 0.001) (Figure 1B). Mean values of difference in PWV for Najuta and non-Najuta were 44 ± 322.0 cm/s (95% CI −35.3 to 123.0) and 204 ± 315.1 cm/s (95% CI 146.7 to 262.1), respectively (Table 2). Individual analysis for each of the non-Najuta devices also showed a significant increase in PWV after surgery: TAG (Before: 2,090 ± 485.9 cm/s vs. After: 2,300 ± 512.1 cm/s, *p* = 0.025, *n* = 20), Relay (Before: 2,102 ± 465.3 cm/s vs. After: 2,206 ± 444.4 cm/s, *p* = 0.004, *n* = 20), Valiant (Before: 1,696 ± 330.2 cm/s vs. After: 2,186 ± 378.7 cm/s, *p* < 0.001, *n* = 30), and Zenith (Before: 2,084 ± 431.7 cm/s vs. After: 2,321 ± 500.6 cm/s, *p* < 0.001, *n* = 47). Mean values of difference in PWV for TAG, Relay, Valiant, and Zenith were 210 ± 447.6 cm/s, 104 ± 157.0 cm/s, 217 ± 345.3 cm/s, and 237 ± 276.9 cm/s, respectively (Figures 2A–D; Table 2).

**Table 2:** Changes in pulse wave velocity after surgery for each device.

	**Before**	**After**	***p***	**Mean of difference**	**SD of difference**	**95% CI**	**Cohen's *d***
Najuta	2,040 ± 346.8 cm/s	2,084 ± 390.5 cm/s	0.136	43.83 cm/s	322 cm/s	−35.31 to 123.0 cm/s	0.14
Non-Najuta	2,058 ± 422.1 cm/s	2,263 ± 462.8 cm/s	<0.001	204.4 cm/s	315.1 cm/s	146.7 to 262.1 cm/s	0.65
TAG	2,090 ± 485.9 cm/s	2,300 ± 512.1 cm/s	0.025	209.6 cm/s	447.6 cm/s	0.126 to 419.1 cm/s	0.47
Relay	2,102 ± 465.3 cm/s	2,206 ± 444.4 cm/s	0.004	103.7 cm/s	157 cm/s	30.2 to 177.2 cm/s	0.66
Valiant	1,969 ± 330.2 cm/s	2,186 ± 378.7 cm/s	<0.001	216.9 cm/s	345.3 cm/s	87.94 to 345.8 cm/s	0.63
Zenith	2,084 ± 431.7 cm/s	2,321 ± 500.6 cm/s	<0.001	237.2 cm/s	276.9 cm/s	155.9 to 318.5 cm/s	0.86

**Figure 3:**
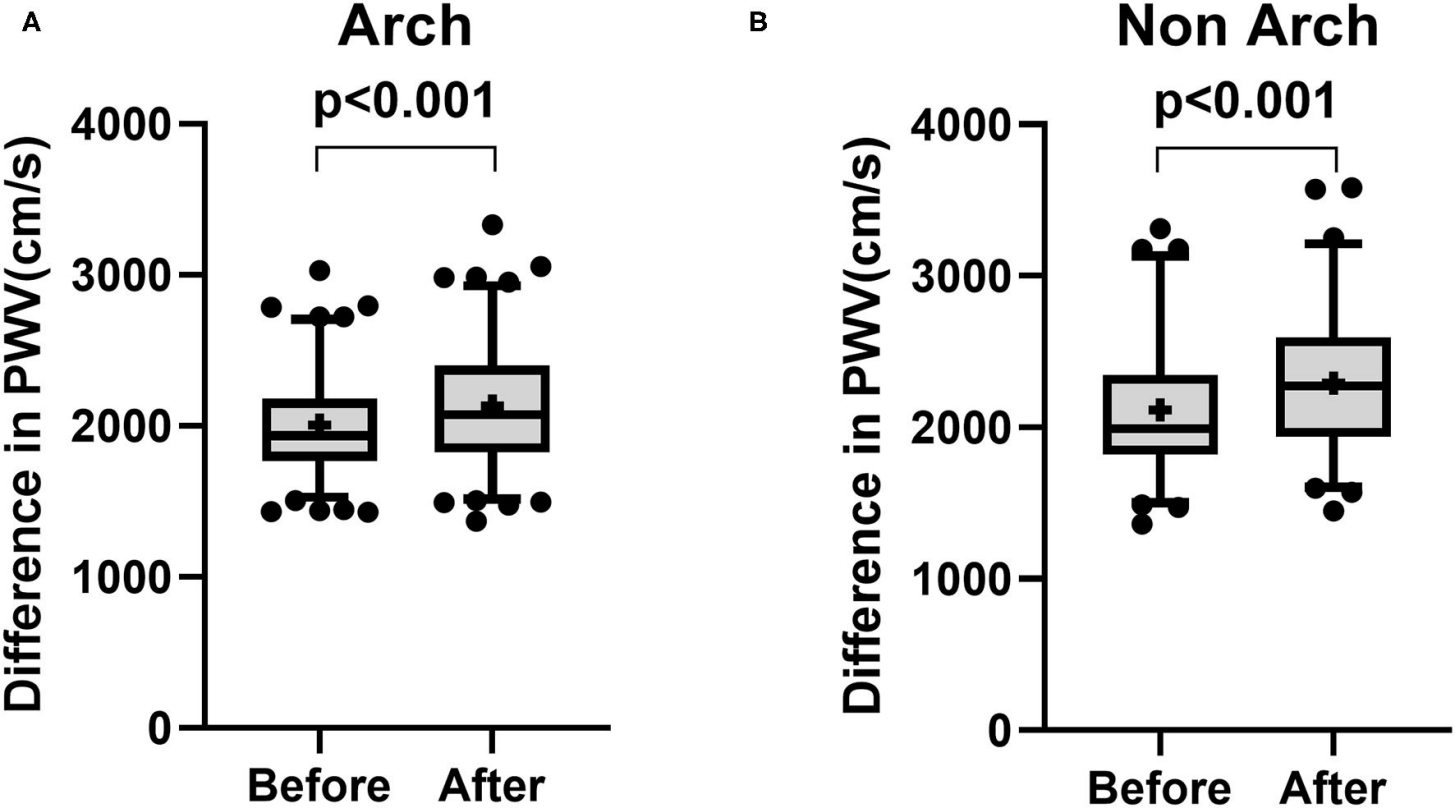
Box and Whisker plot for pulse wave velocity before and after surgery for patients treated from aortic arch (**A**: Arch) and patients treated from descending thoracic aorta (**B**: non-Arch). There was a significant increase in pulse wave velocity in patients treated from aortic arch **(A)** and patients treated from descending thoracic aorta **(B)**. Whisker shows 5–95 percentile range and + shows the mean value.

The demographics of patients treated from aortic arch (Arch) and descending thoracic aorta (non-Arch) are shown in Table 3. Creatinine was higher in patients receiving endoprosthesis from the descending thoracic aorta [Arch: 0.87 mg/dl (0.71–1.21) vs. non-Arch: 1.03 mg/dl (0.82–1.31), *p* = 0.027]. Najuta was more often used in patients treated from the aortic arch (Arch: 55.1% vs. non-Arch: 9.2%, *p* < 0.001), and the treatment index was higher in patients treated from the aortic arch (Arch 1.33 ± 0.376 vs. non-Arch: 1.15 ± 0.297, *p* < 0.001). There was a significant increase in PWV in patients treated from aortic arch (Before: 2,006 ± 333.7 cm/s vs. After: 2,132 ± 423.7 cm/s, *p* < 0.001) and patients treated from descending thoracic aorta (Before: 2,116 ± 460.9 cm/s vs. After: 2,292 ± 460.9 cm/s, *p* < 0.001) (Figures 3A,B). The mean values of difference in PWV before and after surgery for patients treated from aortic arch and descending thoracic aorta were 126 ± 317 cm/s (95% CI 63.97 to 186.5) and 176 ± 338.2 cm/s (95% CI 98.48 to 253.1), respectively (Table 4).

**Table 3:** Demographics of the patients receiving endovascular prosthesis from the aortic arch (Arch) and from the descending thoracic aorta (non-Arch).

	**Arch**	**Non-Arch**	***p***
	***n* = 107**	***n* = 76**	
Age (years)	75 ± 7.9	74 ± 7.7	0.41
Male (%)	79 (73.8%)	60 (78.9%)	0.49
Creatinine (mg/dl)	0.87 (0.71–1.21)	1.03 (0.82–1.31)	0.027
Hemoglobin (g/dl)	12.4 ± 1.73	12.5 ± 1.84	0.56
Hypertension (%)	102 (95.3%)	71 (93.4%)	0.74
Dyslipidemia (%)	51 (47.7%)	34 (44.7%)	0.76
Diabetes (%)	20 (18.7%)	21 (27.6%)	0.21
Chronic obstructive pulmonary disease (%)	11 (10.3%)	12 (15.8%)	0.27
Cerebral vascular disease (%)	18 (16.8%)	10 (13.2%)	0.54
Ischemic heart disease (%)	23 (21.5%)	21 (27.6%)	0.38
Tobacco user (%)	79 (73.8%)	58 (76.3%)	0.73
Angiotensin receptor blocker (%)	39 (36.4%)	30 (39.5%)	0.76
Calcium channel blocker (%)	79 (73.8%)	49 (64.5%)	0.19
Beta blocker (%)	40 (37.4%)	38 (50.0%)	0.1
HMG-CoA inhibitor (%)	52 (48.6%)	40 (52.6%)	0.65
Najuta (%)	59 (55.1%)	7 (9.2%)	<0.001
Treatment length (mm)	214.7 ± 57.63	186.2 ± 48.82	<0.001
Treatment index (mm/cm)	1.33 ± 0.376	1.15 ± 0.297	<0.001
Difference in PWV (cm/s)	125.7 ± 316.99	175.8 ± 338.24	0.31
Difference in sBP (mmHg)	−0.3 ± 13.83	−1.7 ± 16.21	0.53

*sBP, systolic blood pressure*.

**Table 4:** Changes in pulse wave velocity for patients treated for Arch and non-Arch.

	**Before**	**After**	***p***	**Mean of difference**	**SD of difference**	**95% CI**	**Cohen's *d***
Arch	2,006 ± 337.7 cm/s	2,132 ± 423.7 cm/s	<0.001	125.7 cm/s	317 cm/s	64.97 to 186.5 cm/s	0.40
Non-arch	2,116 ± 460.9 cm/s	2,292 ± 460.9 cm/s	<0.001	175.8 cm/s	338.2 cm/s	98.48 to 253.1 cm/s	0.52

**Figure 4:**
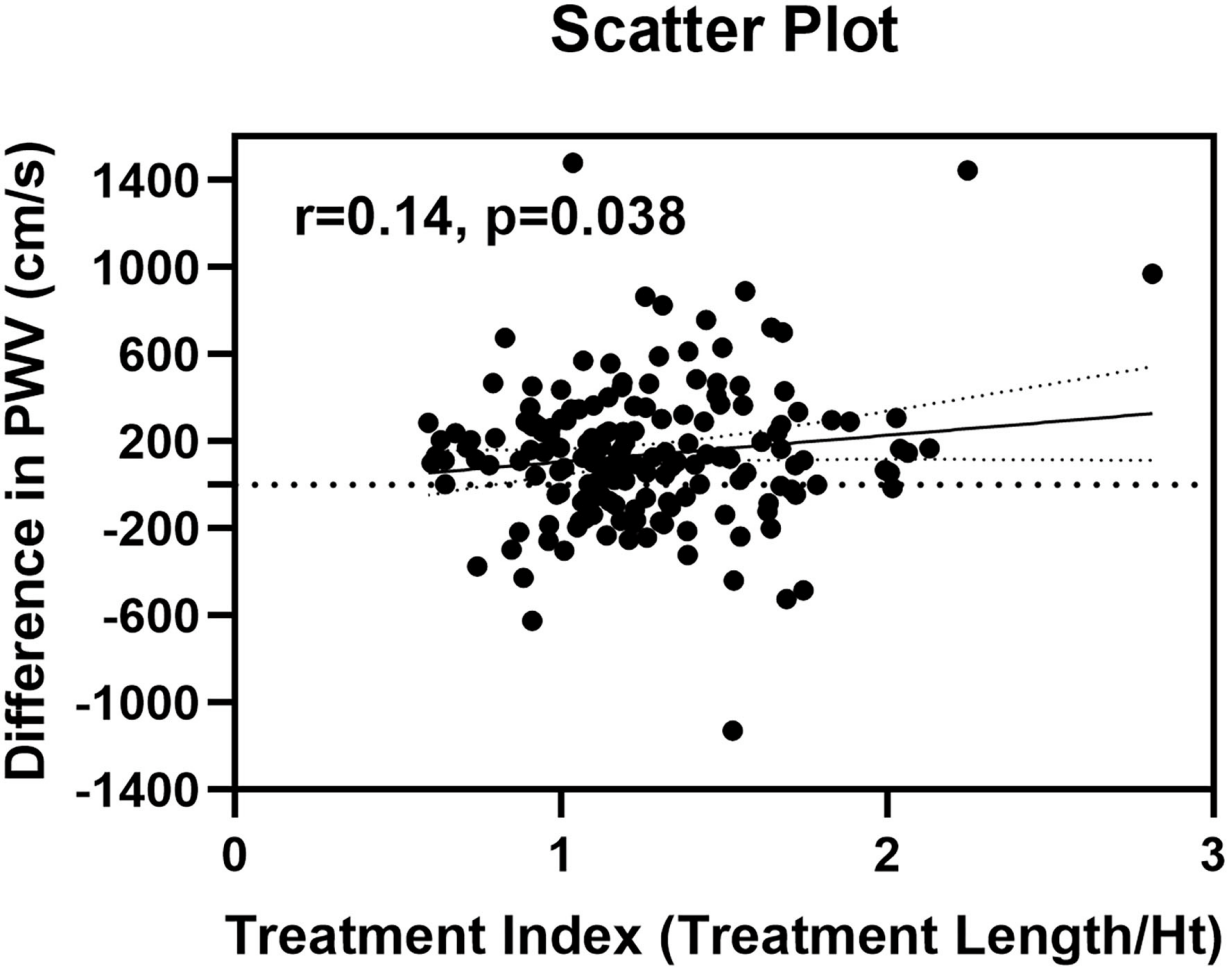
Scatter plot of differences in pulse wave velocity and treatment index.

The scatterplot for treatment length index and changes in PWV showed a significant but weak correlation (*r* = 0.14, 95% CI 0.01 to 1.00, *p* = 0.038) (Figure 4). Furthermore, there were no significant factors associated with the difference in PWV after surgery (Table 5). Multivariate linear regression model for the difference in PWV, including possible confounding factors, showed that angiotensin receptor blocker (Coef 96.28, 95% CI 0.332 to 192.230, *p* = 0.049), use of Najuta (Coef −219.43, 95% CI −322.684 to −116.176, *p* < 0.001), and treatment index (Coef 147.57, 95% CI 24.826 to 270.312, *p* = 0.019) were independent factors associated with changes in PWV (*R*^2^ = 0.324). However, the treatment site was not associated with changes in PWV (Coef 26.02, 95% CI −73.596 to 125.627, *p* = 0.61) (Table 6).

**Table 5:** Changes in pulse wave velocity (cm/s) in patient with or without listed variables.

	**With Najuta**			**Without Najuta**		
	**Without**	**With**	***p***	**Without**	**With**	***p***
Hypertension (%)	27.5 ± 302.99	153.4 ± 326.78	0.24	118.6 ± 240.48	209.9 ± 319.34	0.46
Diabetes (%)	142.6 ± 310.36	159.9 ± 379.20	0.77	198.3 ± 284.60	227.2 ± 414.84	0.69
Dyslipidemia (%)	174.7 ± 379.92	114.0 ± 248.21	0.21	232.8 ± 374.62	170.2 ± 221.97	0.29
Chronic obstructive pulmonary disease (%)	153.1 ± 322.57	96.7 ± 358.60	0.44	207.1 ± 323.83	187.4 ± 261.45	0.82
Cerebrovascular disease (%)	141.9 ± 317.20	171.7 ± 376.51	0.66	187.9 ± 293.93	301.9 ± 416.47	0.17
Ischemic heart disease (%)	159.3 ± 328.06	106.3 ± 319.80	0.35	211.5 ± 335.76	179.8 ± 232.43	0.65
Tobacco user (%)	173.7 ± 361.76	137.4 ± 313.99	0.52	263.1 ± 348.46	183.3 ± 301.55	0.23
Postoperative angiotensin converting enzyme inhibitor (%)	142.6 ± 330.73	201.9 ± 253.79	0.54	200.1 ± 319.06	272.7 ± 253.76	0.56
Postoperative angiotensin receptor blocker (%)	128.8 ± 295.47	196.3 ± 399.02	0.22	179.4 ± 245.61	265.6 ± 440.08	0.18
Postoperative calcium channel blocker (%)	153.8 ± 379.58	140.3 ± 274.35	0.78	245.6 ± 354.03	171.5 ± 278.57	0.21
Postoperative beta blocker (%)	144.5 ± 344.50	148.9 ± 305.31	0.93	207.6 ± 309.77	201.4 ± 322.66	0.92
Postoperative HMG-CoA inhibitor (%)	125.8 ± 368.26	166.1 ± 280.84	0.41	165.6 ± 362.67	235.5 ± 270.08	0.24

**Table 6:** Linear regression model for changes in pulse wave velocity (*R*^2^ = 0.324).

	**Coef**	**95% Confidence interval**	***p***
Age	2.14	−3.247 to 7.528	0.43
Male	−24.96	−124.529 to 74.603	0.62
Angiotensin receptor blocker	96.28	0.332 to 192.230	0.049
Calcium channel blocker	−25.07	−114.348 to 64.215	0.58
Beta blocker	−17.51	−108.373 to 73.348	0.7
HMG-CoA inhibitor	−17.98	−111.145 to 75.183	0.7
Najuta	−219.43	−322.684 to −116.176	<0.001
Treatment index	147.57	24.826 to 270.312	0.019
Arch treatment	26.02	−73.596 to 125.627	0.61
Difference in sBP	10.27	7.355 to 13.188	<0.001

*sBP, systolic blood pressure*.

## Discussion

With improvement in endovascular technology, endovascular treatment of aortic aneurysm has currently become a class IIa indication for descending thoracic aortic aneurysm (Hiratzka et al., 2010; Erbel et al., 2014). The outcomes of thoracic endovascular treatment are also promising, especially in high-risk patients. In a study with 579 patients undergoing endovascular treatment of descending thoracic aorta, in-hospital mortality, stroke, and spinal cord injury occurred in 4.7, 2.1, and 0.5% of the patients, respectively. For the long-term outcomes, the aorta-specific survival at 11.8 years was 96.2% (Ranney et al., 2018). Previous reports on commercially available devices have also shown an acceptable outcome with a major advance event rate of 5.6% in patient receiving Relay within 1 year after surgery; 2.8% in patient requiring secondary intervention within 1 year with Cook device; a major adverse event rate of 7.6% in patient receiving cTAG within 4 years after surgery (Bodell et al., 2018). Characterized by its minimal invasiveness and versatility, more young patients are now treated by this technology. Although prevention of aortic rupture is the main goal, physiological changes after implantation of endoprosthesis could be a major concern, especially in young patients with expected long-term survival.

A previous study has shown a significant increase in PWV after thoracic endovascular aortic repair in *ex vivo* porcine model (de Beaufort et al., 2017). In an experiment using a pulsatile mock loop system, cTAG, Relay, Zenith, and Valiant showed a significant increase in PWV after implantation. For the total cohort of 20 aortas, PWV increased by a mean of 600 cm/s or 8.9% of baseline PWV after deployment of a 100-mm endoprosthesis (*p* < 0.001). This change was more profound than that observed in our study. This study showed that in a clinical setting, the treatment length of 192 ± 56.28 mm in patients receiving non-Najuta devices (cTAG, Relay, Valiant, and Zenith) was associated with a mean increase of 204 ± 315.1 cm/s in PWV after surgery. These findings may imply that complex regulation of hemodynamics in humans may be attenuating the effect of implanted endoprosthesis on physiological changes in the aorta.

Previously, we have reported changes in PWV in patients undergoing endovascular treatment of aortic arch aneurysm, which increased from 2,083 ± 454.5 cm/s before surgery to 2,305 ± 479.7 cm/s after surgery (*p* = 0.023) (Hori et al., 2020). Furthermore, an increase in PWV in patients undergoing thoracic endovascular aortic replacement was associated with long-term cardiovascular events and cerebrovascular events in Kaplan–Meyer analysis (Hori et al., 2020). PWV has been reported to be a useful biomarker for major adverse cardiovascular and cerebrovascular events (MACCE) and mortality (Sutton-Tyrrell et al., 2005). A meta-analysis of cohort studies has shown that a 1 m/s increase in PWV was associated with a 12% increase in the risk of cardiovascular events (Munakata, 2016). This study has shown that the mean difference in PWV after surgery was 204 ± 315.1 cm/s (95% CI 146.7 to 262.1) for patients receiving non-Najuta devices. The result from a previous study may indicate that these patients receiving non-Najuta devices may be associated with a 2.4% (12% × 204 cm/s/1,000 cm/s) increase in the risk of cardiovascular events. In contrast, the mean increase in PWV of 44 cm/s in patients receiving Najuta may not be of clinical significance. Medications have also been reported to reduce PWV, which include antihypertensive drugs, statins, peroral antidiabetics, advanced glycation end products (AGE) cross-link breakers, anti-inflammatory drugs, endothelin-A receptor antagonists, and vasopeptidase inhibitors (Janic et al., 2014). Although these medications have shown some effects in reducing PWV, their effects on long-term outcomes are still to be evaluated. Furthermore, their effects on patients receiving endoprosthesis are another factor that should be recognized. In this study, angiotensin receptor blocker was associated with an increase in PWV. This may be due to the background of patients receiving these medications, including hypertension and ischemic heart disease, which are the factors associated with increased PWV (Sutton-Tyrrell et al., 2005; Munakata, 2016; Tomiyama et al., 2016).

This study and previous study from our group have shown that there was no significant change in PWV in patients receiving Najuta (Hori et al., 2017, 2020). There are several types of endovascular devices in the market depending on the differences in the fabric used and stent skeleton design. Comparison of expanded polytetrafluoroethylene (ePTFE) and dacron graft on PWV has shown that ePTFE graft has less effect on the changes in PWV after surgery (Kadoglou et al., 2014). This phenomenon was also observed in the computational analysis (Kleinstreuer et al., 2008). In a study by Kleinstreuer et al. (2008), the wall distensibility of a stent graft compliance (C) was calculated by the difference in maximum diameter during systolic pressure and diastolic pressure (Δ*d*) over pulse pressure (Δ*p*) and the diastolic diameter of the vessel (*d*):


$$\begin{array}{l} \text{C}=\Delta \text{d}/\Delta \text{pd}\left(\%/100mmHg\right) \end{array}$$

The compliance of a diamond nitinol stent with an ePTFE graft was 1.7%–2.0%/100 mmHg compared with 0.1–0.2%/100 mmHg with a dacron graft. TAG is constructed with an ePTFE, while Relay, Valiant, and Zenith are constructed with a dacron graft. Furthermore, all of these devices are exoskeleton type stent graft in which the graft fabric is covered by stent skeleton. On the contrary, Najuta is characterized by endoskeleton type stent graft with ePTFE graft. The graft is only sutured at the edge of the stent skeleton, and thus, ePTFE graft is able to stretch out with the pulsatile flow. Sail-like effect of the graft fabric in Najuta may have substituted the physiological effect of the aortic compliance, thus reducing the effect on PWV. Furthermore, the stent skeleton of Najuta is rather compliant compared with other devices. Device selection may be an important factor in providing less effect on PWV after surgery. Further advancement in the skeleton design and fabric to accommodate for healthy aortic compliance may provide better long-term outcomes.

Through the Windkessel effect, the aorta stores 50% of left ventricular volume during systole, which is released at diastole by its elastic force (Kanzaki, 2014). This phenomenon allows the constant flow to the peripheral, which reduces mechanical stress on the aorta. It has also been reported that the aortic arch contributes ~50% of the total arterial compliance (Ioannou et al., 2009; Kanzaki, 2014). Therefore, the aortic compliance changes in the aortic arch may have a more profound effect on the changes in PWV compared with other sites of the aorta. However, multivariate analysis of this study has shown that treatment site was not an independent factor associated with an increase in PWV. In contrast, despite the weak correlation between the treatment length index and changes in PWV, the multivariate linear regression model has shown that the treatment length index was an independent factor associated with an increase in PWV after normalizing for possible confounding factors. In a previous study using *ex vivo* porcine model, de Beaufort et al. have shown that an increase in PWV was dependent on the extent of stent coverage, resulting in an increase by a mean of 1,400 cm/s or 23.0% of baseline PWV after an extension of covered length (*p* < 0.001) (de Beaufort et al., 2017). Although these changes were more profound than those observed in our previous study, this study has shown a similar result in the clinical setting, in which the treatment length was associated with the degree of changes in PWV after surgery. The result of this study showed that for a patient with a height of 170 cm, the treatment length of 170 mm would increase PWV by 147.57 cm/s (95% CI 24.826 to 270.312). Adjustment of the treatment length could range from 20 to 40 mm, and thus modification in surgical strategy may not provide clinical significance. However, changes in PWV after endovascular treatment should be recognized as a mean increase in PWV was observed at a range of 44 to 237 cm/s with commercially available endoprostheses.

### Limitations

There are several limitations. First, this is a retrospective study with a relatively small sample size. Further study with a larger sample size is recommended to confirm these results. Second, PWV measurement within 1 year of surgery was evaluated in this study. PWV at a longer period after surgery should also be evaluated in which remodeling of the aorta or thrombotic changes in the aneurysm sac may have an effect on PWV. There are few patients with significant aortic remodeling, which shows the disappearance of an aortic aneurysm in long term. The aortic compliance of these patients may be reflected by the characteristics of the implanted endoprosthesis. However, in patients with residual thrombosed aneurysm sac, the movement of the endoprosthesis with the pulsatile flow may be limited by the thrombosed tissue surrounding the endoprosthesis. Finally, further study with the evaluation of long-term outcomes should be performed to observe whether postoperative changes in PWV reflect long-term outcomes. The present cohort showed a trend toward higher preoperative PWV in patients with ischemic heart disease (IHD: 2,109 ± 466.1 cm/s vs. no IHD: 1,996 ± 449.9 cm/s, *p* = 0.15). This already may imply that patients with higher PWV may be associated with cardiovascular events, and thus an increase in PWV after surgery may put patients at higher risk for cardiovascular events as reported in the previous reports (Vlachopoulos et al., 2012; Tomiyama et al., 2016). In our hospital, every patient is evaluated for coronary artery disease prior to surgery. Patients with significant stenosis of the coronary artery were treated prior to surgery or immediately after the surgery. This could be an important bias that could affect the long-term outcomes in our cohort. Despite these limitations, there are strong evidence that increased PWV is associated with major cardiovascular and cerebral vascular events (Sutton-Tyrrell et al., 2005; Mattace-Raso et al., 2006; Munakata, 2016; Tomiyama et al., 2016; Ohkuma et al., 2017; Takae et al., 2019). Changes in PWV after surgery should be recognized as a possible risk factor for the long-term cardiovascular events.

## Conclusion

This study showed that the Najuta device may provide less effect on PWV in patients undergoing endovascular treatment of the thoracic aortic aneurysm. There was no significant difference in changes in PWV for different treatment sites. Although modifying endovascular treatment may not provide clinical significance, treatment length was associated with an increase in PWV after implantation of endoprostheses.

## Data Availability Statement

The original contributions presented in the study are included in the article/supplementary material, further inquiries can be directed to the corresponding author/s.

## Ethics Statement

The studies involving human participants were reviewed and approved by Institutional review board of Saitama Medical Center, Jichi Medical University. Written informed consent for participation was not required for this study in accordance with the national legislation and the institutional requirements.

## Author Contributions

DH and TF: had substantial contributions to the conception or design of the work. DH: acquisition. DH, TF, SK, and TY: analysis. DH, NK, and AY: interpretation of data for the work and revision of the work. DH, TF, SK, and TY: drafting of the work. All authors approved the final version of the work.

## Funding

This work was supported by JSPS KAKENHI [Grant Number JP21K16500 (DH)] from the Ministry of Education, Culture, Sports, Science and Technology, Japan.

## Conflict of Interest

AY serves as a consultant to Japan Lifeline Co. Ltd., which was a distributor for Relay device. The remaining authors declare that the research was conducted in the absence of any commercial or financial relationships that could be construed as a potential conflict of interest.

## Publisher's Note

All claims expressed in this article are solely those of the authors and do not necessarily represent those of their affiliated organizations, or those of the publisher, the editors and the reviewers. Any product that may be evaluated in this article, or claim that may be made by its manufacturer, is not guaranteed or endorsed by the publisher.
